# Deep Learning Framework for Liver Segmentation from *T*_1_-Weighted MRI Images

**DOI:** 10.3390/s23218890

**Published:** 2023-11-01

**Authors:** Md. Sakib Abrar Hossain, Sidra Gul, Muhammad E. H. Chowdhury, Muhammad Salman Khan, Md. Shaheenur Islam Sumon, Enamul Haque Bhuiyan, Amith Khandakar, Maqsud Hossain, Abdus Sadique, Israa Al-Hashimi, Mohamed Arselene Ayari, Sakib Mahmud, Abdulrahman Alqahtani

**Affiliations:** 1 NSU Genome Research Institute (NGRI), North South University, Dhaka 1229, Bangladesh; 2 Department of Electrical Engineering, Qatar University, Doha 2713, Qatar; 3 Department of Computer Systems Engineering, University of Engineering and Technology Peshawar, Peshawar 25000, Pakistan; 4 Artificial Intelligence in Healthcare, IIPL, National Center of Artificial Intelligence, Peshawar 25000, Pakistan; 5 Center for Magnetic Resonance Research, University of Illinois Chicago, Chicago, IL 60607, USA; 6 Hamad Medical Corporation, Doha 3050, Qatar; 7 Department of Civil Engineering, Qatar University, Doha 2713, Qatar; 8 Department of Medical Equipment Technology, College of Applied, Medical Science, Majmaah University, Majmaah City 11952, Saudi Arabia; 9 Department of Biomedical Technology, College of Applied Medical Sciences, Prince Sattam Bin Abdulaziz University, Al-Kharj 11942, Saudi Arabia

**Keywords:** deep learning, automated liver segmentation, MRI, diagnostic radiology, *T*1-weighted contrast

## Abstract

The human liver exhibits variable characteristics and anatomical information, which is often ambiguous in radiological images. Machine learning can be of great assistance in automatically segmenting the liver in radiological images, which can be further processed for computer-aided diagnosis. Magnetic resonance imaging (MRI) is preferred by clinicians for liver pathology diagnosis over volumetric abdominal computerized tomography (CT) scans, due to their superior representation of soft tissues. The convenience of Hounsfield unit (HoU) based preprocessing in CT scans is not available in MRI, making automatic segmentation challenging for MR images. This study investigates multiple state-of-the-art segmentation networks for liver segmentation from volumetric MRI images. Here, T1-weighted (in-phase) scans are investigated using expert-labeled liver masks from a public dataset of 20 patients (647 MR slices) from the Combined Healthy Abdominal Organ Segmentation grant challenge (CHAOS). The reason for using T1-weighted images is that it demonstrates brighter fat content, thus providing enhanced images for the segmentation task. Twenty-four different state-of-the-art segmentation networks with varying depths of dense, residual, and inception encoder and decoder backbones were investigated for the task. A novel cascaded network is proposed to segment axial liver slices. The proposed framework outperforms existing approaches reported in the literature for the liver segmentation task (on the same test set) with a dice similarity coefficient (DSC) score and intersect over union (IoU) of 95.15% and 92.10%, respectively.

## 1. Introduction

Over the past decade, remarkable advancements in deep learning (DL) algorithms have led to a rapid transformation in the field of radiology. DL-aided diagnostics have achieved exceptional accuracy in detecting abnormalities in various domains such as ophthalmology, respiratory, and breast imaging. In some cases, multimodal DL solutions now exhibit accuracy levels comparable to expert radiologists. The high performance and clinically satisfactory outcomes achieved through computer-aided diagnostic radiology were previously considered inconceivable [1,2,3].

Semantic segmentation is a prerequisite for any DL-driven diagnostic task, as it allows the model to learn from the region of interest. Formerly, for semantic segmentation tasks in radiology/medical imaging, distinct mathematical models were implemented, but such approaches often lacked a generalized solution. Deep learning-based segmentation tasks outperform conventional mathematical modeling-based approaches. Segmentation has always helped in improving the performance of computer-aided diagnosis [4,5]. Q. Dou et al. present a unique 3D deeply supervised network (3D DSN) explicitly designed for liver segmentation from CT data [6]. The network incorporates deep supervision to enhance optimization and discrimination capabilities during the learning process, resulting in competitive segmentation results compared to state-of-the-art approaches, along with improved processing speeds. In another study, C. Chen et al. propose an innovative method for lung lesion segmentation in CT scans of COVID-19 patients. Their approach involves region-of-interest extraction and employs a 3D network with attention mechanisms to enhance segmentation accuracy [7]. Additionally, C. Chen et al. introduce a rapid and precise lung segmentation technique, utilizing the edge-weighted random walker algorithm with spatial and clustering information to achieve a heightened accuracy and reduced segmentation time [8]. Similarly, P. Hu et al. develop a liver segmentation framework by integrating a 3D convolutional neural network (CNN) with globally optimized surface evolution. Their approach demonstrates effective segmentation outcomes suitable for clinical applications [9]. Together, these contributions significantly enhance the field of automated organ segmentation, offering valuable insights for medical imaging research and clinical implementations.

However, such a segmentation task in an anatomical paradigm, i.e., the identification and delineation of an anatomical area or structure in magnetic resonance imaging (MRI), encounters a colossal amount of complexity. The complexity can be due to topology, spatial distance, location, relative motion, texture, geometrical structure, and other varying anatomical information. As a consequence, anatomical segmentation has always been a demanding task. In particular, compared to other anatomical structures, very few significant works can be found that focus on liver segmentation [10,11,12].

For any deep learning-based liver disease diagnosis system, precise automated liver segmentation is indispensable. However, similar to any anatomical segmentation task, it is extensively challenging. This is due to the fact that, compared with other abdominal organs, its anatomy can noticeably differ with patients and clinical conditions. Additionally, the liver’s proximity to contiguous abdominal organs (the spleen and kidneys) generates substantial ambiguity [13,14].

However, recent research has demonstrated excellent results for deep neural network (DNN)-based liver segmentation tasks from volumetric abdominal computed tomography (CT) images. Tang et al. [15] achieved a dice similarity coefficient (DSC) of 98% in the liver segmentation task from a plain CT scan using a modified multiscaled convolutional neural network (CNN). Hu et al. [9] used a three-dimensional CNN for the same task and achieved a high performance of around a 97.25% dice similarity coefficient. These works utilized Hounsfield unit (HoU) scaling as a hyperparameter for image enhancement in the preprocessing stage [16]. The review by Xiang et al. [17] observed that, in terms of liver segmentation from magnetic resonance imaging (MRI) scans, high performance could not be achieved and also very little significant work exists in this domain. Owing to the absence of such homogeneous HoU-based image enhancement convenience, in terms of automated liver segmentation from volumetric abdominal MR scans, achieving a similarly high performance to CT images is challenging.

Moreover, MRI scans are extensively adopted by clinicians for liver pathology investigation, due to their superior contrast and spatial resolution for soft tissues compared to CT scans [18,19]. CT scans can provide solid anatomical information. On the contrary, MRI demonstrates high signal intensity in comparison to CT scans. As a result, both anatomical and physiological information can be derived from MRI scans. In particular, both CT and MRI can provide accurate anatomical information about the liver lesion or haemangioma; however, MRI scans can provide a further important basis for screening benign or malignant types [20,21,22].

The above studies demonstrate the significance of liver segmentation, specifically from volumetric MRI scans, as this modality is favored by clinicians in relation to pathological diagnosis. In this regard, liver segmentation from MRI scans holds significant importance. A. Mostafa et al. investigated a whale optimization algorithm for liver segmentation from MRI scans [23]. A. Hänsch et al. studied multimodal training and three-dimensional CNN for the task [24]. X. Zhong et al. used deep action learning with a 3D UNet [25], and P. Pandey et al. investigated contrastive semisupervised learning for the liver segmentation task [26] on the CHAOS abdominal MRI dataset [27]. D. Mitta et al. implemented a weighted UNet with attention gates for the liver segmentation task [28] on the same dataset, and J. Hong et al. achieved a slightly better performance using a source-free unsupervised UNet [29]. X. Wang et al. investigated a bidirectional search of the neural net for the task [30]. Additionally, S. Mulay et al. used a geometric edge enhancement-based mask R-CNN [31]. The more recent work of L. Zbinden et al. achieved better performance than previous research for liver segmentation on the same testing set by implementing nnUNet on $T_{1}$-weighted MRI slices [32].

In this research, we investigated 24 state-of-the-art segmentation networks for liver segmentation tasks from $T_{1}$-weighted MR scans using a publicly available dataset, which annotated ground truths for the liver segmentation of 20 patients. The prospect of predicting a precise mask from $T_{1}$-weighted MR scans is higher as fat (and protein) contents are brighter and more distinguishable in such a group. The investigation explores state-of-the-art segmentation networks, such as UNet, UNet++, and feature pyramid network (FPN) segmentation networks with varying dense encoder backbones, along with various image enhancement techniques in the preprocessing stage. The proposed cascaded network showed superior performance to many high-performance state-of-the-art approaches on the same test set. Finally, we developed a software prototype by deploying our proposed DL model in a cloud server for public usage. The cross-platform software is open source and can be accessed from http://130.211.209.103/projects/the-big-mri-project-beta, accessed on 9 August 2023.

The main contributions of the research are listed below.

- This research extensively investigates state-of-the-art approaches for precise liver segmentation from T1-weighted abdominal MR scans to facilitate clinicians with AI-driven assistance for liver pathology diagnosis;
- This research investigates the effects of multiple image enhancement techniques for automated liver segmentation tasks from MR scans;
- This research proposes a novel cascaded network for the liver segmentation task that demonstrated state-of-the-art performance compared to the literature;
- The proposed model was deployed in a cloud server for demonstration purposes so that clinicians can directly benefit from the results of this investigation.

## 2. Materials and Methods

**Figure 1:**
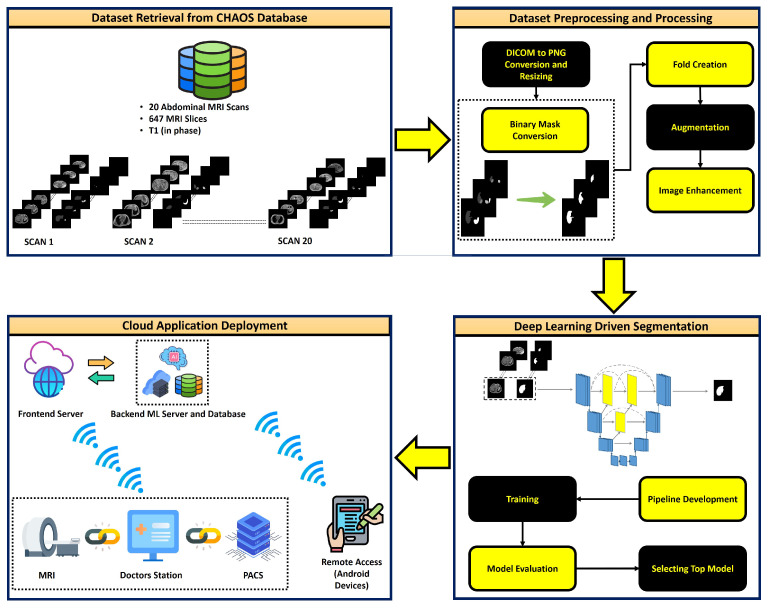
Flow diagram explaining methodology for automated liver segmentation from $T_{1}$-weighted MRI scans.

The brief methodology of the research is explained in Figure 1. The methodology will be discussed in detail in the following section.

### 2.1. Dataset

The dataset was collected from the Combined Healthy Abdominal Organ Segmentation (CHAOS) grant challenge [27]. The public portion of the CHAOS dataset includes computed tomography (CT) and magnetic resonance imaging (MRI) abdominal scans of 20 patients, in the Digital Imaging and Communications in Medicine (DICOM) format. The ground truths (GT) were provided from the source, which includes masks for the right kidney, left kidney, liver, and spleen. The ground truth masks were annotated by certified radiologists. All scans are of healthy patients. The MRI scans include $T_{1}$-weighted in-phase and out-phase, along with $T_{2}$-weighted scans, which are discussed in the next subsection. Each $T_{1}$ scan includes 26 to 56 slices; for 20 patients the total number of $T_{1}$-weighted slices is 647 [27].

### 2.2. Selecting Task-Specific Contrast Group

Among different contrast-enhanced groups (in-phase $T_{1}$-weighted, out-phase $T_{1}$-weighted, and $T_{2}$-weighted), specific groups were chosen by analyzing the relevant abdominal anatomy and attributes of the available contrast-enhanced groups.

#### 2.2.1. Relevant Abdominal Anatomy

**Figure 2:**
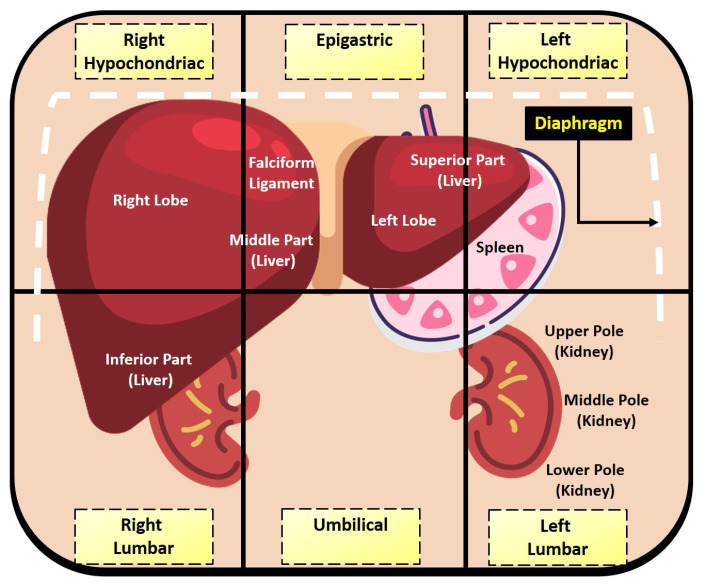
Superficial visualization of relevant abdominal anatomy for describing underlying ambiguity in the segmentation task.

The supplied masks (left kidney, right kidney, liver, and spleen) lie in close proximity to each other in the abdominal region, leading to a colossal amount of ambiguity in distinguishing any of the organs. The anatomy of these organs is briefly visualized in Figure 2 [33]. The superior part of the liver (left lobe) lies within the epigastric and left hypochondriac regions. It is in close proximity to the spleen and rests in front of the spleen in terms of the axial plane. The middle part of the liver resides above the umbilical region. The inferior part of the liver is just in front of the upper pole of the right kidney, which occupies the right lumbar region. The left kidney lies in the left lumbar region just below the spleen. Therefore, such close proximity generates an enormous amount of complexity and obscurity in automated abdominal organ segmentation tasks using machine learning.

#### 2.2.2. $T_{1}$- and $T_{2}$-Weighted Images

The $T_{1}$ and $T_{2}$ parameter represents relaxation time for longitudinal ($M_{z}$) and transverse ($M_{t}$) magnetization components for each proton. $T_{1}$ is noted as the spin–spin relaxation phenomenon, and $T_{2}$ is noted as the spin–lattice relaxation phenomenon. When the macroscopic magnetizing vector for each voxel is $M_{o}$, then the relationship among the magnetization components and $T_{2}$, $T_{1}$ is denoted as [34]

(1) $$M_{t}\left(t\right)=M_{o}\sin\alpha e^{\frac{-t}{T_{2}}}$$

(2) $$M_{z}\left(t\right)=M_{o}\cos\alpha e^{\frac{-t}{T_{1}}}+M_{o}\left(1-e^{\frac{-t}{T_{1}}}\right)$$

where α denotes the flip angle, which represents a rotation in net magnetization. Characteristically, the $T_{1}$ tissue relaxation time is always larger than $T_{2}$. The relaxation times vary broadly with tissue attributes and characteristics. These varying intervals can also be used to distinguish between healthy and abnormal tissues. Table 1 denotes $T_{1}$ and $T_{2}$ values for relevant abdominal tissues [35].

The relation among the image intensity of each voxel I(x,y), the tissue density ρ(x,y), the echo time (TE), and the repetition time (TR) can be denoted as

(3) $$I\left(x,y\right)=\rho \left(x,y\right)\frac{(1-e^{\frac{TR}{T_{1}}})\sin\alpha }{1-e^{\frac{TR}{T_{1}}}\cos\alpha }e^{\frac{TE}{T_{2}}}$$

In Equation (3), α is optimized by following

(4) $$\alpha_{Ernst}=\cos^{-1}e^{\frac{TR}{T_{1}}}$$

when $TE\ll T_{2}$, and either $\alpha ∼\alpha_{Ernst}$ or $TR∼T_{1}$, then the image is defined as $T_{1}$-weighted. Moreover, the image is defined as $T_{2}$-weighted when $TE>T_{2}$, and either $\alpha \ll \alpha_{Ernst}$ or $TR\gg T_{1}$ [36].

**Figure 3:**
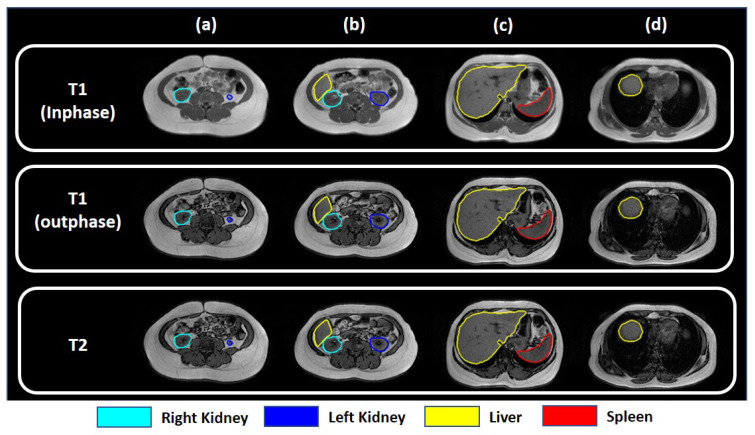
Visualization of MRI slices from (**a**) upper pole of the kidney, (**b**) inferior part of the liver, (**c**) middle part of the liver, and (**d**) superior part of the liver for different types of data available in the dataset.

In accordance with its definitions, fat (and protein) content in $T_{1}$-weighted MRI scans is brighter. Owing to such characteristics, the liver is more distinguishable in $T_{1}$-weighted MRI scans. Figure 3 shows sample $T_{1}$- and $T_{2}$-weighted slices of different axial views. It is clear from the figure that for $T_{1}$-weighted in-phase scans, the liver is far more distinguishable (even in the slices where the liver is small) in the inferior part of the liver, and the superior part of the liver in the axial view. In the MRI slices where the liver is larger (i.e., the middle part of the liver), both in-phase and out-phase $T_{1}$-weighted scans can be used. As $T_{1}$-weighted out-phase scans represent out-of-phase protons, a darker boundary can be noticed around regions of varying intensities. As a result, unwanted artifacts are introduced in these slices.

Due to such attributes among different contrast-enhanced MRI scans, in-phase $T_{1}$-weighted contrast-enhanced scans were selected for the liver segmentation task. Such groups provide initially enhanced images, which can contribute to boosting the performance of deep neural networks.

### 2.3. Dataset Preprocessing

Firstly, in-phase $T_{1}$-weighted 647 DICOM slices were converted to PNG format in order to optimize the preprocessing and processing steps. In the ground truth mask, there are multiple organs (right kidney, left kidney, liver, and spleen) present. Binary masks are generated for the liver alone. Each slice and GT mask pair is then resized to 256×256 dimensions from their original 512×512 dimensions. Reducing the size of the dataset offers notable benefits in terms of enhancing computational efficiency during the training process of segmentation networks.

**Figure 4:**
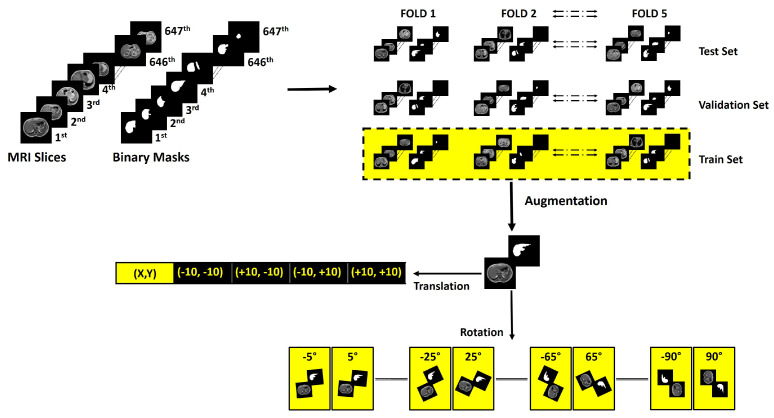
Flow diagram explaining the methodology for fold creation and augmentation in the training set.

**Figure 5:**
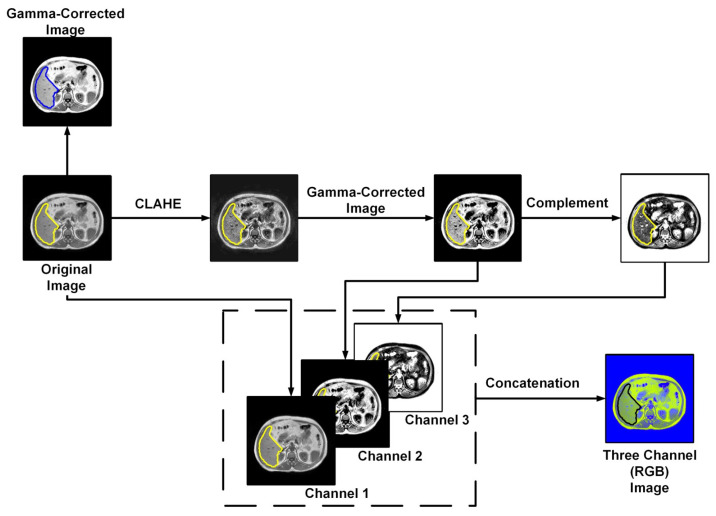
Visualization of image enhancement techniques.

In order to ensure the data are ready for the machine learning investigation, there are important steps that include fold creation from the preprocessed dataset. Fold creation invovles dividing the data into the training set, validation set, and testing set for five folds. In order to avoid biases during training, it is important to make sure that the dataset is balanced; this is achieved by the augmentation of the training set. Finally, the authors investigated different image enhancement techniques for each of the created folds. Figure 4 represents techniques for fold creation and augmentation, which performed following the literature in [37,38,39]. Image enhancement techniques are demonstrated in Figure 5.

#### 2.3.1. Fold Creation

The methodology follows five-fold cross-validation techniques for validating the network performances. From the preprocessed dataset, five folds were created. In each fold training, validation, and testing set ratios were 70%, 10%, and 20%, which corresponds to 453, 65, and 129 DICOM slices, respectively. This was done to make sure that the performance metric represents the performance of the trained network on the complete dataset.

#### 2.3.2. Augmentation

The training set for each fold was augmented using geometrical spatial transformation of coordinates (rotation and translation). Geometric spatial transformations represent a widely recognized and efficient technique for processing topographic imaging datasets [40,41].

The affine matrix for rotation $I_{rotation}$ and for translation $I_{translation}$ can be denoted as

(5) $$I_{rotation}=\left[\begin{array}{ccc} \cos\theta & \sin\theta & 0\\ -\sin\theta & \cos\theta & 0\\ 0 & 0 & 1 \end{array}\right]$$

(6) $$I_{translation}=\left[\begin{array}{ccc} 1 & 0 & 0\\ 0 & 1 & 0\\ t_{x} & t_{y} & 1 \end{array}\right]$$

where the values of θ are defined by the set:

(7) θ={±5°,±10°,±15°,±20°,…,±90°}

and the values $\left(t_{x},t_{y}\right)$ are defined by the set:

(8) $$\left(t_{x},t_{y}\right)=\left\{\left(-10,10\right),\left(+10,-10\right),\left(-10,+10\right),\left(10,10\right)\right\}$$

The validation and testing sets were not augmented. After augmentation, each training fold consisted of around 6700 slices. The validation set was used to avoid overfitting, which is a common problem in machine learning model development [42,43].

#### 2.3.3. Image Enhancement

Image enhancement includes gamma correction for each fold. For each of the pixels f(x,y), the gamma correction can be denoted as [44]

(9) $$g\left(x,y\right)=255{\left(\frac{f(x,y)}{255}\right)}^{\frac{1}{\lambda }}$$

where g(x,y) denotes the gamma corrected pixel value, and the value of λ is considered to be 0.5 in this study. And, for all f(x,y)>200, f(x,y) is considered to be 255 to enhance the targeted region. Another image enhancement technique called contrast-limited adaptive histogram equalization (CLAHE) was used in the three-channel (or RGB) image construction technique. If in a histogram kth, the intensity value is $r_{k}$, and the number of pixels with the $r_{k}$ intensity value is $n_{k}$, then for an M×N dimensional image, the equalized histogram can be represented by

(10) $$p\left(r_{k}\right)=\frac{n_{k}}{M\times N}$$

CLAHE is an adaptive histogram equalization technique that undergoes transformation over local regions. Here, a matrix of 8×8 dimension was used for local histogram equalization. The output histogram from the CLAHE transformed image follows the Rayleigh distribution. Gamma correction was applied to the CLAHE-enhanced image, and finally, the image was complemented. The image compliment $f^{-1}\left(x\right)$ can be expressed as

(11) $$f^{-1}\left(x\right)=255-f\left(x\right)$$

The three-channel (or RGB) image was constructed by concatenating the original image, the gamma-corrected CLAHE enhanced image, and the complement of the gamma-corrected CLAHE enhanced image.

### 2.4. Deep Neural Networks

**Figure 6:**
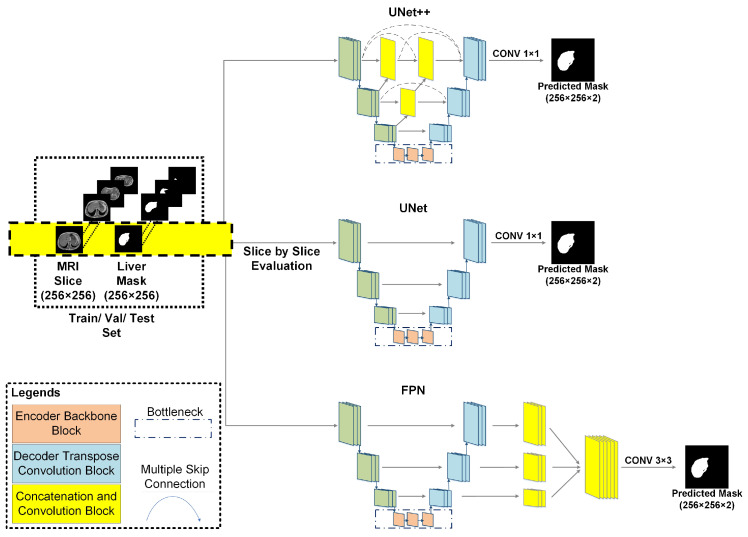
Network architectures of different segmentation networks and investigation frameworks. The varying depth of pretrained dense, residual, and inception encoder backbones were investigated for UNet++, UNet, and FPN segmentation network architectures.

UNet-like architectures with pretrained deep dense, residual, and inception encoder backbones previously showed high performance in both classification and segmentation tasks for 2D chest X-rays [45]. UNet++ with deep dense blocks showed benchmark performance in segmenting lung content from volumetric CT scans [46]. These segmentation networks also performed well in solving complex problems such as detecting intracranial hemorrhages [47]. These studies inspired us to investigate these UNet-like architectures with pretrained encoder backbones for liver segmentation tasks from MR scans. UNet, UNet++, and feature pyramid network (FPN) segmentation networks were investigated with varying depths of dense, residual, and inception encoder backbones. The network architectures are shown in Figure 6. The encoder backbones were pretrained dense, residual, and inception blocks (marked in light orange). The decoder (light blue) uses transpose convolution blocks for upscaling the vector output from the bottleneck (marked in dark blue) output to construct the segmentation mask. The yellow blocks in UNet++ and FPN represent both concatenation and convolution blocks.

#### 2.4.1. UNet

UNet architecture consists of an encoder and a decoder part. The encoder part reduces the input image size in each of the convolutional blocks through max pooling. In the final encoder block, the two-dimensional original image matrix is reduced to a vector array. The decoder part upscales the converted vector array in each block through convolutional blocks and upconvolution layers. Lastly, the skip connections among encoder–decoder blocks transfer weights for localizing the region of interest. These skip connections are similar to the attention mechanism [48].

#### 2.4.2. UNet++

UNet++ is an extension of the UNet and wide UNet architecture. It utilizes the concept of deep supervision. UNet++ also introduces nested convolutional blocks inside each skip pathway, and such blocks enhance the quality of feature spaces that are passed to the decoder blocks [49,50].

#### 2.4.3. Feature Pyramid Network (FPN)

In the FPN network, weight connections from the UNet decoder blocks are fed through skip connections to feature pyramid blocks. Further, the output from each feature pyramid block is fed into a single convolutional block. Finally, the output from the convolutional block is fed into a rectilinear unit (ReLU) activation layer for generating the predicted masks [51].

#### 2.4.4. Pretrained Backbones

The concept of transfer learning is utilized to enhance the segmentation performance and reduce the training time. Several pretrained encoders (variants of dense, residual, and inception networks), which were trained on the ImageNet computer vision database [52], were used as the backbones. For each backbone variant, three varying depths were investigated. The variants of DenseNets were DenseNet201, DenseNet161, and DenseNet121 [52,53,54], while the variants of residual networks were ResNet152, ResNet50, and ResNet18 [55]. InceptionV4 and InceptionResNet were the variants of the inception backbones [56].

### 2.5. Experiments

Two major experiments were carried out in this study: (i) the generalized model and (ii) the specialized network for handling anatomical ambiguity.

#### 2.5.1. Generalized Model

In this experiment, MRI slices with different liver sizes were used in training and evaluation and the model was generally not specific to any particular liver size. Then, the effects of image enhancement on the generalized model were investigated. A total of 24 networks (three architectures with eight backbones) were tested on three versions of MRI images (i.e., original, gamma-corrected, and 3-channel view) to segment the liver.

#### 2.5.2. Specialized Network for Handling Anatomical Ambiguity

To enhance the performance of the segmentation network in segmenting the liver region from the MRI slices where the liver shape varies, multiple segmentation networks needed to be trained to segment the liver region reliably. A total of 90 slices from the inferior part of the liver and the upper right pole of the kidney were trained separately. Exact preprocessing and processing steps were followed for this set of slices, which was discussed previously. For this specific task, only varying depths of the ResNet encoder–decoder backbones with UNet++, UNet, and FPN were investigated as the ResNet showed better performance in the preliminary study. Three variants of ResNet and Inception-ResnetV2 with three architectures (a total of 12 experiments) were investigated specifically for the slices with small liver contents.

#### 2.5.3. Cascaded Network

**Figure 7:**
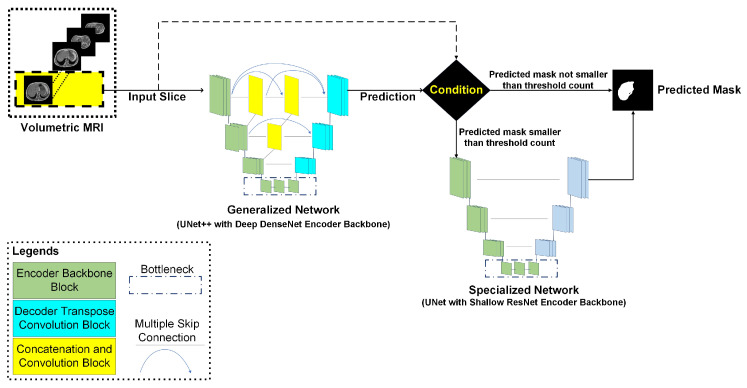
Cascaded network for handling anatomical ambiguity: the generalized network predicts an initial mask; if the pixel count for the predicted mask refers to a constrained or null liver content, then the input slice is fed into the specialized network.

Since the liver size varies in the MRI volume, every single generalized model proposed in the literature fails to generalize. Therefore, we propose a cascaded model using a decision function to improve the performance of the segmentation network. The architecture of the cascaded network is depicted in Figure 7. The volumetric MRI scan is fed into the network slice by slice. At first, a liver mask is predicted from the generalized network. From the first predicted mask, the number of predicted white pixels is calculated. The following equation is used to decide the potential shape of the liver mask in the slice under investigation, where k represents the white pixel count:

(12) $$Liver\text{\_}Content=\left\{\begin{array}{c} Absent,\;\mathrm{if}\;k=0\\ Small,\;\mathrm{if}\;1\leq k\leq 750\\ large,\;\mathrm{if}\;k>750 \end{array}\right.$$

If the number of white pixels is zero, there is no liver in the slice and so the mask is completely black. However, if the decision function identifies a number between 1–750, the slice is again fed into the specialized network for producing the final mask. However, if the number is higher than 750, the mask generated by the generalized model is used as the final liver mask.

The sets of large, small, and absent liver contents are created on the basis of the topographic visualization of the abdominal anatomy, which was described earlier in Section 2.2.1. The liver content is maximum in the axial views from the middle part of the liver. Moreover, the liver content is medium and constrained in the axial views from the superior part of the liver and the inferior part of the liver, respectively. In the axial view from the upper part of the kidney, the liver content is absent. In this perspective, the set of large liver content is constructed with the axial views from the middle part and superior part of the liver. The axial views from the inferior part of the liver are represented in the set of small liver content. Lastly, the set of absent liver content is formed by the axial views from the upper part of the right kidney. The threshold values are then determined by analyzing the pixel counts in each of the sets.

Generally, the axial views from the superior part and inferior part of the liver have significant liver content and the liver area can be comfortably segmented. However, ambiguity arises for the axial views from the inferior part of the liver and the upper part of the right kidney, as the liver portion is significantly constrained. In such a perspective, segmentation performance may improve if slices from these two complicated axial views are handled with a separate network, which is only trained with such cases. Thus, such a cascaded approach was investigated.

### 2.6. Loss Function

Binary cross-entropy (BCE) loss is typically used for classification tasks. As any semantic segmentation task can be considered as a classification task at the pixel level, this loss is also effective for segmentation. BCE loss can be expressed by [57,58]

(13) $$Loss_{\left(BCE\right)}=\frac{1}{N}\sum_{i=0}^{N-1}-\left(y_{i}\log\left({\hat{y}}_{i}\right)+\left(1-y\right)\log\left(1-{\hat{y}}_{i}\right)\right)$$

The dice coefficient is used to calculate the similarity index between ground truth and predicted masks for segmentation tasks. Dice loss is a region-based loss function and it is introduced in [59]. Dice loss can be expressed by

(14) $$Loss_{\left(DICE\right)}=1-\frac{\sum_{i=0}^{N-1}y_{i}{\hat{y}}_{i}}{\sum_{i=0}^{N-1}y_{i}^{2}+\sum_{i=0}^{N-1}{\hat{y}}_{i}^{2}+\epsilon }$$

In Equations (13) and (14), *N* represents the total number of pixels, $y_{i}$ represents the *i*th pixel in the ground truth mask, and ${\hat{y}}_{i}$ represents the *i*th pixel

Initially, both the mentioned loss functions were investigated to find the optimum solution. However, the detailed investigation was carried out with the BCE loss, as it demonstrated superior performance over dice loss in the initial investigation.

### 2.7. Training Parameters

In order to conduct a uniform comparison among the network performances, it was indispensable to use the same training parameters for all the networks. All the training was conducted in an NVIDIA Tesla P100-PCIE graphics processing unit (GPU) with 16 gigabytes (GB) og memory. The initial learning rate was set to 0.0001 with a learning factor LR of 0.02. If the validation loss did not show significant changes in 10 epochs, the learning rate was reduced by $\frac{1}{LR}$. The maximum epoch number was set to be 100 for each fold, but if the validation loss was constant for 20 epochs, the training was terminated. *Z*-score normalization was used, which uses the standard deviation and mean of the raw MRI slices for normalizing each image. For optimizing the process of gradient descent, in each of the epochs, the ADAM optimization algorithm was used as it showed superior performance over stochastic gradient descent (SGD) in the initial investigation [60,61].

### 2.8. Evaluation Metrics

For evaluating the performance of each investigated network, the accuracy, dice similarity coefficient (DSC) (i.e., F1-score), and intersection of union (IoU) were computed. For each network, the average metrics for each ground truth mask and predicted mask pairs were calculated. Accuracy, DSC, and IoU can be expressed by

(15) $$Accuracy=\frac{TP+TN}{2\times TP+TN+FP+FN}$$

(16) $$DSC=\frac{2\times TP}{2\times TP+FP+FN}$$

(17) $$IoU=\frac{TP}{2\times TP+FP+FN}$$

In Equations (15), (16), and (17), TP, TN, FP, and FN denote true positive, true negative, false positive, and false negative, respectively.

### 2.9. Cloud Deployment

A cloud-based application for real-time liver segmentation from MRI images was deployed. The deep learning model was deployed in the cloud back-end server, which runs on an 8-core, 32 GB Memory Apache Linux instance hired from Google Cloud Perform (GCP). The back-end server was connected to a SQL database for storing the MRI images. The application is cross-platform compatible and users can access the application anytime via a web browser from any edge device. The cloud-based application can be remotely connected with a Picture Archiving System (PACS) for assisting radiologists in liver pathology investigations. In order to provide more convenient remote access for clinicians, an Android application was also developed. Figure 1 superficially describes the cloud application. To ensure the robustness of the segmentation network, an automated self-learning scheduler was implemented in the back-end server following the concept discussed [62]. The scheduler automatically retrains the deployed model with the incoming new data provided by the user, and such an approach boosts the network’s performance on unseen real-world data.

## 3. Results and Discussion

The results from different investigations are discussed in this section. Later, a performance comparison is presented, which compares the efficiency of the proposed approach with the reported high-performance techniques in the literature for liver segmentation on the same MRI test set. In the following section, the performance of the generalized model, specialized model, and cascaded models are presented.

### 3.1. Generalized Model

The Table 2 summarizes the network performance for segmenting the liver region in the MR slices using a generalized model. Deep networks showed superiority in performance in comparison to shallow networks. On the nonenhanced images (original image), UNet++ with dense backbones showed the top performance. UNet++ with a DenseNet201 backbone showed the best performance with a DSC and IoU of 94.3% and 91.00%, respectively. On the nonenhanced images, UNet with different DenseNet backbones exhibited a similar performance.

For the three-channel image set, networks with DenseNet backbones demonstrated slightly better performance compared to other networks. Among the variants of the DenseNet model, DenseNet161 performed the best for this specific image set. Both FPN and UNet with DenseNet161 backbones achieved a DSC of over 93%. Among the investigated image enhancement techniques, the gamma-enhanced image set performed the worst.

**Figure 8:**
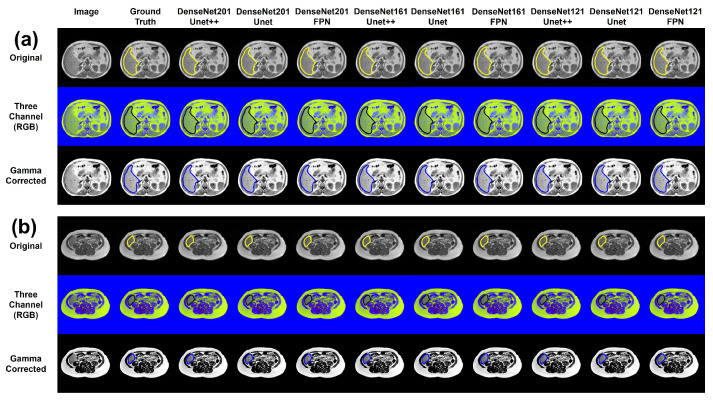
Visualization of the predicted masks from the networks with DenseNet backbones for a sample axial slice showing the middle part of the liver (**a**) and the inferior part of the liver (**b**).

The liver content is maximum in the slices showing the middle part of the liver and also significantly larger in the superior part of the liver. For these two specific types of slices, all of the DenseNet backbones showed excellent performances. Figure 8a shows the predicted liver masks for the slices from the middle part of the liver. The figure shows that all of the networks can segment the liver region accurately.

### 3.2. Effects of Image Enhancement for Generalized Model

It can be observed that the network performances were slightly decreased when image enhancement techniques were implemented (Table 2). This is due to the ambiguity that arises from the slices where the liver content varies widely. Though image enhancement was very effective for the slices where the liver portion was significant, the performance dropped when the liver size was minimum in the slice of investigation. Figure 8b shows such a sample liver slice with the ground truth mask and the masks predicted by different models.

Due to this ambiguity, finding a generalized image enhancement technique for such a complex and varying anatomy is very challenging.

### 3.3. Limitation of the Generalized Model

**Figure 9:**
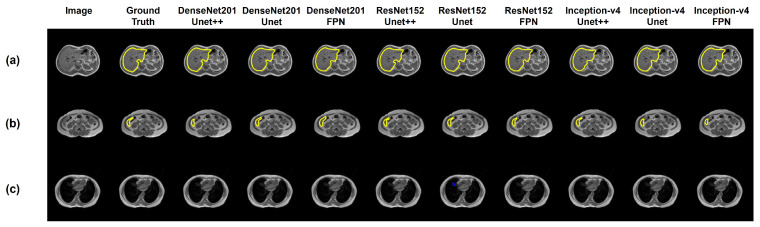
Visualization of the predicted masks from selected networks for a sample slice in (**a**) middle part of the liver (large liver content), (**b**) inferior part of the liver (small liver content), and (**c**) upper pole of the kidney (no liver content).

In the case of the slices of the middle part of the liver, all ResNet and inception backbones demonstrated satisfactory performance. Figure 9 shows the predicted masks from top-performing networks for the middle part of the liver (large liver content), inferior part of the liver (small liver content), and upper pole of the kidney (no liver content) for the original $T_{1}$-weighted images. Figure 9 shows that the generalized model performed well for the slices with large liver content and for the slices where the liver was absent. However, when the liver content was small, the generalized model struggled to locate the liver area precisely.

A more detailed picture is shown in Table 3. The table illustrates the fold-wise slice distribution and the observed DSC for each of the different groups of liver shapes for the top-performing UNet++ model with the DenseNet201 backbone. Though the percentage of slices from the middle part of the liver was the minimum for all the folds, the DSC value for the best-performing model was still over 95% for each fold. Slices with medium liver content occurred the most and the DSC value for each fold was around 95%. The network also efficiently handled slices where the liver content is absent.

However, the model performance was greatly reduced for the slices where liver content was small. It is worth mentioning that the number of such slices in the training set was also insignificant. Our hypothesis was that handling such slices by a separate model may improve the overall segmentation performance, which is explored in this study.

### 3.4. Specialized Network for Handling Anatomical Ambiguity

Table 4 illustrates the performance of the specialized models trained with different architectures and different backbones in comparison to the best-performing generalized model in segmenting the MR slices with a small liver content.

The UNet with ResNet18 encoder backbone showed superior performance over the other investigated networks, with an IoU and DSC of 77.00% and 86.22%, respectively. For UNet++, the Inception-resnet-V2 encoder backbone showed better performance over the varying depths of ResNet backbones with an IoU and DSC of 75.58% and 84.03%, respectively. The shallow ResNet18 backbone performed better for the FPN architecture over other pretrained encoder backbones with an IoU and DSC of 71.20% and 82.04%, respectively. Each of the top-performing encoder backbones for UNet, UNet++, and FPN performed better than the top-performing generalized network for this task, which demonstrated an IoU and DSC of 70.74% and 80.88%, respectively. Lastly, the shallow networks performed better compared to deep networks for this specific task, as the liver content in the slice was small.

### 3.5. Cascaded Network

**Figure 10:**
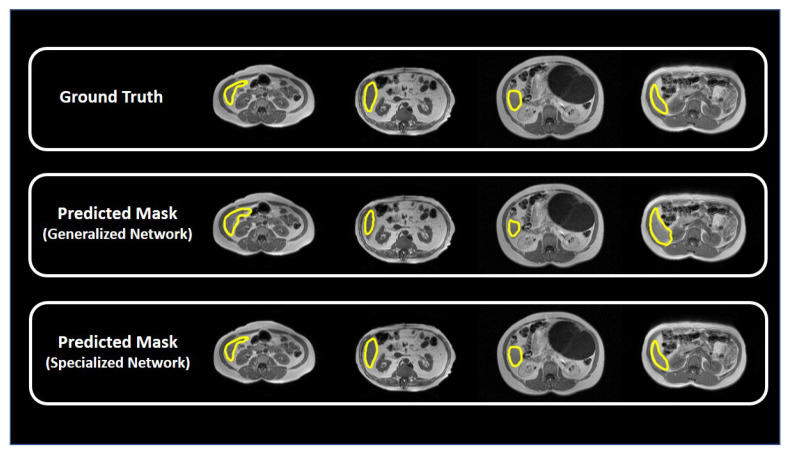
Comparison of the predicted masks from the generalized and specialized networks for sample MR slices with small liver content.

Figure 10 shows the predicted masks from the proposed cascaded network. It can be observed that such an approach enhances the mask quality for slices with a small liver content. Combining both the generalized and specialized network enhances the performance of the network for segmenting the liver region. Table 5 summarizes the performance metrics for the generalized network and the cascaded network. Cascading both the networks improves the overall DSC score (from 94.3 to 95.15%).

### 3.6. Discussion

The performance comparison of our proposed framework with all of the existing high-performance networks (using the same test for evaluation) is summarized in Table 6. X. Zhong et al. [25] investigated deep action learning for abdominal organ segmentation tasks from volumetric MRI images. Their proposed network demonstrated superiority over 3D UNet in terms of overall performance, and achieved a DSC of 80.6% for the liver segmentation task. P. Pandey et al. [26] explored a contrastive semisupervised approach for the same task, and it achieved a DSC of 85.9%. The proposed method generates patches for each slice, which enhances the feature space. Mitta et al. [28] achieved a DSC of 88.12% on the test set by using W-Net with attention gates. J. Hong et al. [29] and X. Wang et al. [30] used source-free unsupervised learning and bidirectional searching for the segmentation task, respectively. By using geometric edge enhancement, S. Mulay et al. [31] boosted the performance of the mask R-CNN for the liver segmentation task on this test set. L. Zbinden et al. [32] achieved a DSC of 93.60% by implementing nnUNet on $T_{1}$-weighted MRI slices.

Our proposed cascaded framework outperforms all of these existing high-performance techniques by a large margin with a DSC of 95.15%. As discussed previously, the size of the liver content in an arbitrary MRI slice depends on its axial view source. Any generalized segmentation network can perform comparatively better when the liver content is significant in the given MRI slice (axial view from the middle part of the liver). On the contrary, the network faces ambiguity when the liver content is reduced for the given MRI slice (axial view from the upper pole of the kidney, the inferior pole of the kidney, and the superior part of the liver). As a result, the network performance drops significantly for these specific groups of slices where the liver content is small. This specific cause for reduced segmentation performance is overlooked in all of the previous studies. Our proposed framework separately handles this specific group of slices with a small liver content, which generates ambiguity through a specialized network, thus enhancing the overall segmentation performance.

## 4. Conclusions

Abdominal organ segmentation is a challenging task due to the complexity of the anatomy of the abdominal area and the close proximity of multiple organs. Ambiguity in the segmentation of the liver arises due to the variance in its anatomical shape in the MRI volume. The MRI modality is favored by clinicians for liver pathology diagnosis. However, automated liver segmentation from MRI scans is a demanding task. In this research, we proposed a novel cascaded network for liver segmentation from $T_{1}$-weighted MR images. The proposed network treats each axial view distinctly and achieved a DSC of 95.15% on the publicly available CHAOS MRI dataset. Such an approach can also be investigated for other abdominal organ segmentation tasks, such as those involving the kidneys and spleen. The proposed network was also deployed as an open-source application in a cloud server for demonstration purposes. This application can later be integrated with PACS for clinical usage. Lastly, we also investigated the effects of different image enhancement techniques for liver segmentation tasks from MR scans.

## Figures and Tables

**Table 1:** Average $T_{1}$ and $T_{2}$ relaxation time (msec) for 1.5 T and 3.0 T MRI scans.

Tissue	1.5 T		3.0 T	
	$T_{1}$ **(msec)**	$T_{2}$ **(msec)**	$T_{1}$ **(msec)**	$T_{2}$ **(msec)**
Kidney	966–1412	85–87	1142–1545	76–81
Liver	586	46	809	34
Spleen	1057	79	1328	79
Lipid	343	58	382	68

**Table 2:** Summary of the investigated network performances from the generalized approach. UNet++ with DenseNet201 encoder exhibited the best performance.

Networks		Original			Three Channel			Gamma Corrected		
**Architecture**	**Backbone**	**Acc.** (%)	**IoU** (%)	**DSC** (%)	**Acc.** (%)	**IoU** (%)	**DSC** (%)	**Acc.** (%)	**IoU** (%)	**DSC** (%)
UNet++	DenseNet201	99.73	91.00	94.30	99.60	88.95	92.35	99.42	89.28	91.91
	DenseNet161	99.68	89.78	93.06	99.71	89.60	92.95	99.66	87.00	90.30
	DenseNet121	99.43	89.17	92.57	99.66	90.08	93.40	99.56	87.58	90.92
	ResNet152	99.70	89.79	93.13	99.67	87.97	91.34	99.70	89.00	92.33
	ResNet50	99.70	90.42	93.81	99.68	89.54	92.98	99.66	88.13	91.64
	ResNet18	99.70	89.73	93.08	99.70	89.63	93.01	99.63	84.55	88.50
	Inception-resnet-v2	99.71	89.16	92.57	99.65	88.31	92.10	99.70	89.60	91.98
	inception-v4	99.70	87.98	91.29	99.68	89.23	92.62	99.70	89.79	92.16
UNet	DenseNet201	99.76	89.98	93.22	99.72	88.77	92.13	99.78	88.74	92.18
	DenseNet161	99.57	90.48	93.84	99.43	90.08	93.60	99.45	87.58	90.92
	DenseNet121	99.43	89.88	93.27	99.66	89.48	92.90	99.64	87.04	90.31
	ResNet152	99.69	89.46	92.97	99.67	88.66	92.25	99.67	88.91	92.35
	ResNet50	99.68	87.48	90.93	99.66	85.49	89.01	99.68	88.79	92.36
	ResNet18	99.67	88.16	91.83	99.67	86.83	90.38	99.68	88.77	92.31
	Inception-resnet-v2	99.66	87.68	91.41	99.68	88.20	91.80	99.70	87.81	91.32
	inception-v4	99.68	88.64	92.34	99.70	90.68	93.47	99.62	87.89	91.71
FPN	DenseNet201	99.65	89.45	92.83	99.36	89.50	92.97	99.47	88.33	91.87
	DenseNet161	99.70	89.38	92.77	99.66	89.32	93.00	99.53	88.11	91.85
	DenseNet121	99.47	87.52	91.08	99.47	89.39	92.94	99.71	86.91	90.49
	ResNet152	99.66	88.49	92.08	99.67	88.90	92.59	99.68	87.85	91.46
	ResNet50	99.69	89.01	92.52	99.65	88.15	91.88	99.66	88.76	92.40
	ResNet18	99.68	88.33	91.91	99.66	88.10	91.95	99.67	88.58	92.33
	Inception-resnet-v2	99.61	87.03	91.46	99.67	88.17	92.08	99.65	88.52	92.39
	inception-v4	99.62	85.52	90.85	99.66	88.64	92.55	99.66	88.64	92.55

**Table 3:** Distribution of slices of distinct axial views in train set and test set, along with the observed DSC. Slices depicting axial view from the inferior part of the liver holds a constrained liver content and exhibits anatomical ambiguity.

Fold No	Middle Part of Liver (Liver Content: Large)			Superior Part of Liver (Liver Content: Medium)			Inferior Part of Liver (Liver Content: Small)			Upper Part of Kidney (Liver Content: Absent)		
	Train Set Slice %	Test Set Slice %	DSC (%)	Train Set Slice %	Test Set Slice %	DSC (%)	Train Set Slice %	Test Set Slice %	DSC (%)	Train Set Slice %	Test Set Slice %	DSC (%)
1	7.74%	19.38%	97.03%	45.63%	34.89%	95.33%	12.71%	16.28%	81.95%	34.12%	29.46%	100.00%
2	7.73%	11.63%	95.75%	44.36%	41.86%	95.11%	13.25%	11.63%	82.64%	34.66%	34.88%	95.55%
3	6.69%	17.83%	96.17%	43.43%	41.86%	95.20%	11.79%	12.40%	78.13%	35.85 %	30.23%	97.43%
4	7.67%	10.85%	95.70%	43.26%	42.63%	95.23%	14.90%	9.30%	80.20%	34.18%	37.21%	97.91%
5	6.86%	16.79%	97.35%	44.25%	38.17%	93.90%	14.15%	9.16%	82.46%	34.75%	35.88%	94.78%

**Table 4:** Summary of the investigated network performance for the slices with small liver content using different specialized models and the best-performing generalized model. UNet with ResNet18 backbone showed improved performance for the task.

Networks		Metrics (Specialized Network)			Metrics ( Best-Performing Generalized Network)		
**Architecture**	**Backbone**	**Acc.**(%)	**IoU** (%)	**DSC** (%)	**Acc.** (%)	**IoU** (%)	**DSC** (%)
UNet	ResNet18	99.64	77.00	86.22			
	ResNet50	99.81	72.06	80.94			
	ResNet152	99.78	70.00	79.73			
	Inception-resnet-v2	99.70	72.73	81.72			
UNet++	ResNet18	99.78	71.71	78.38			
	ResNet50	99.76	71.62	80.96			
	ResNet152	99.80	71.89	81.02	99.76	70.74	80.88
	Inception-resnet-v2	99.78	75.58	84.03			
FPN	ResNet18	99.80	71.20	82.04			
	ResNet50	99.77	69.75	79.77			
	ResNet152	99.78	71.86	81.20			
	Inception-resnet-v2	99.80	70.92	80.41			

**Table 5:** Performance metrics for the best-performing generalized and cascaded network. Here, the results of the cascaded network are marked in gray.

Experiments	Acc. (%)	IoU (%)	DSC (%)
Generalized Network	99.73%	91.00%	94.30%
Cascaded Network	99.70%	92.10%	95.15%

**Table 6:** Comparison of the proposed method (marked in gray) with existing studies that used the same testing set.

Authors	Methodology and Approach	Metric (DSC)
X. Zhong et al. [25]	Deep action learning with 3D UNet	80.60 ± 5.30%
P. Pandey et al. [26]	Contrastive Semi Supervised Learning Approach with UNet	85.90%
D. Mitta et al. [28]	W-Net with attention gates	88.12%
J. Hong et al. [29]	Source Free Unsupervised UNet	88.40%
X. Wang et al. [30]	Bidirectional Searching Neural Net	89.80%
S. Mulay et al. [31]	Mask R-CNN	80.00%
	Geomatric Edge Enhancement based Mask R-CNN	91.00%
L. Zbinden et al. [32]	nnUNet	93.60%
Proposed	Cascaded Network for Handling Anatomical Ambiguity	95.15%

## Data Availability

This article is based on previously conducted studies and does not contain any studies with human participants or animals. The dataset used in this research is open access and can be accessed from https://chaos.grand-challenge.org/Data/, accessed on 9 August 2023. Kavuar et al. [27] followed standard protocol for creating this open-access dataset.
